# A Rare Case of Back Pain in a 4 Month-Old Baby with Type A Haemophilia

**DOI:** 10.5334/jbsr.1804

**Published:** 2019-07-24

**Authors:** Dimitar Boyadzhiev, Dana Dumitriu

**Affiliations:** 1 Cliniques Universitaires St-Luc, Brussels, BE

**Keywords:** Haemophilia, spinal epidural hematoma, ultrasound

A four-month-old baby with previous history of type A hemophilia was admitted to our institution for minor head trauma, after falling on his back from a sitting position. The physical examination, as well as non-contrast head computed tomography (CT) and spine X-ray performed in the emergency room were all normal.

**Figure 1:**
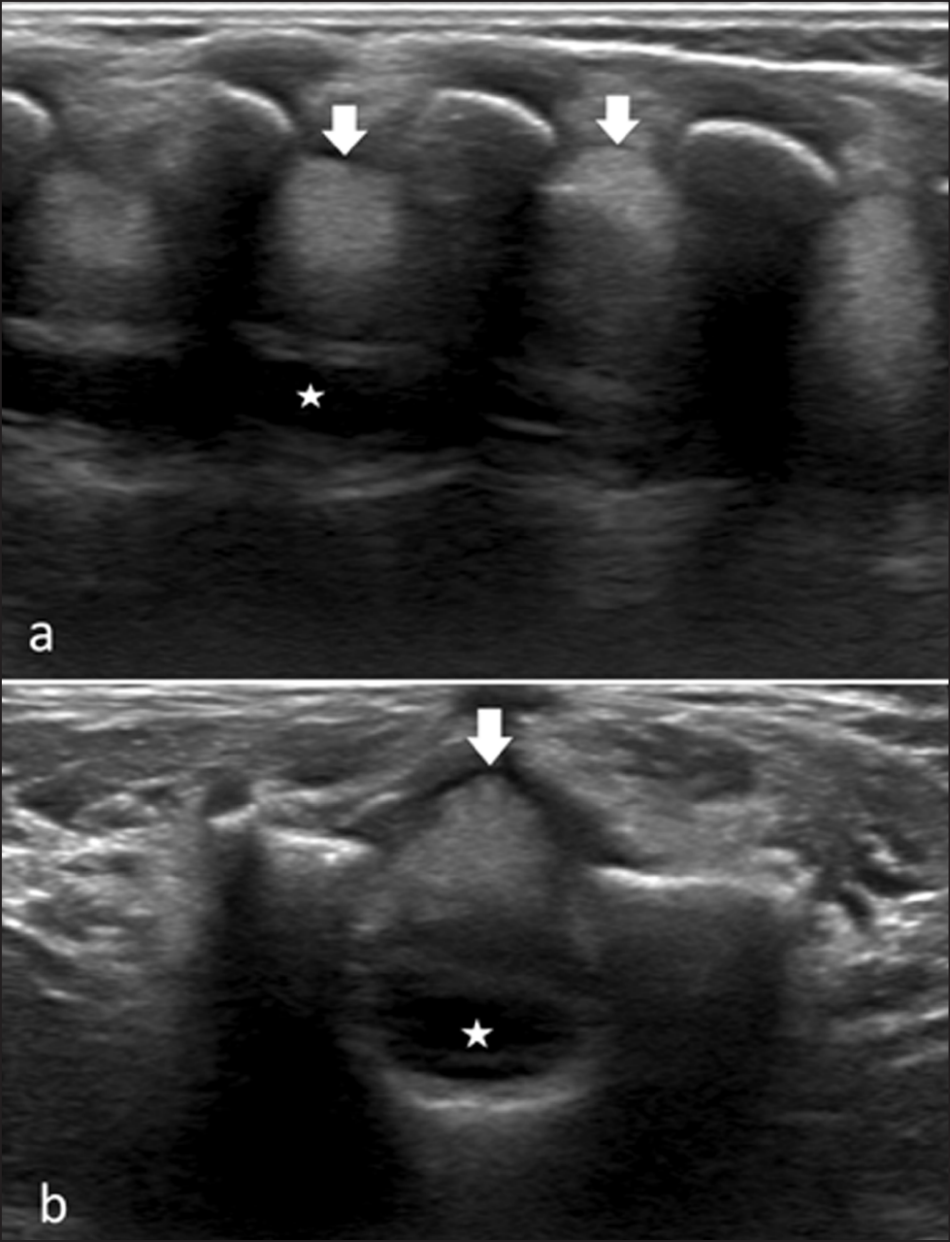


**Figure 2:**
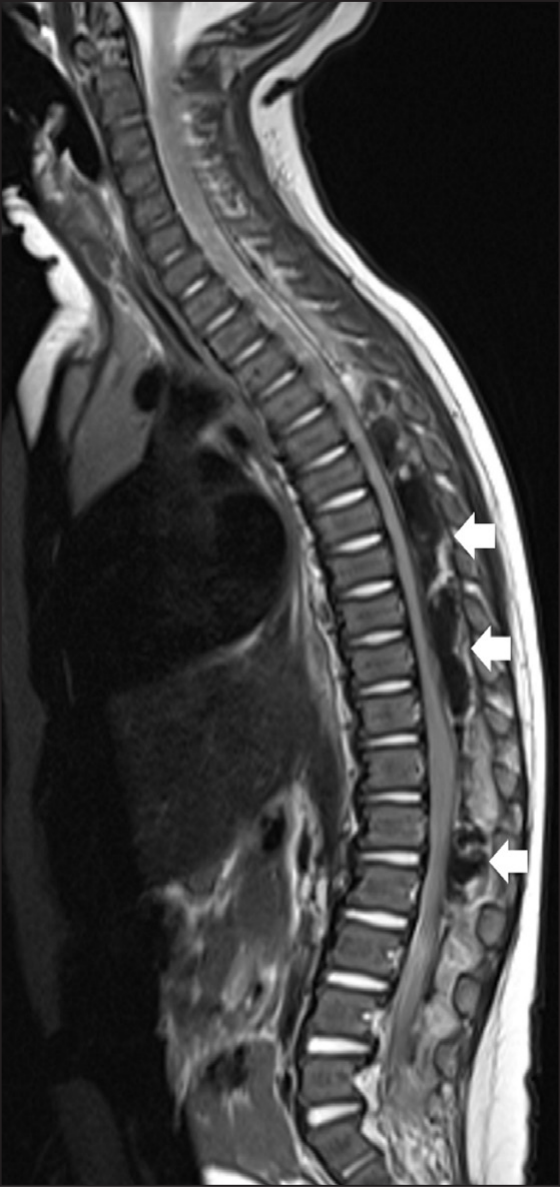


The following day, the child developed a low-grade fever and back pain and grew more restless. In search of a spinal complication, an ultrasound of the spinal canal was performed. It revealed a hyperechoic heterogeneous mass behind the spinal cord, extending in the lower dorsal and lumbar regions (arrows in Figure 1 a, b). Mass effect with anterior shift of the spinal cord (* in Figure 1 a, b) was identified. Given the medical history of the child and the ultrasound findings, a diagnosis of extradural spinal hematoma was suggested. Emergency spinal magnetic resonance imaging (MRI) was performed to evaluate the full extension and mass effect of the hematoma and assess the need for surgical decompression. MRI revealed that the hematoma occupied the length of the entire spinal canal, from the cervical (C1) to the sacral (S1) level (Figure 2). It compressed the spinal cord, with the maximum mass effect situated at mid-dorsal level (T7–T8). Emergency surgical drainage of the hematoma was performed, with decompression of the spinal cord. Replacement therapy with factor VIII was started before surgery. The surgical outcome was good and the child recovered completely.

Hemophilia A is an X-linked recessive disorder caused by factor VIII deficiency and accounts for 85% of hemophilic cases [1]. Spinal epidural hematoma (SEH) is a rare complication from hemophilia and represents 2–8% of all central nervous system hemorrhages. Only one other case within the same age range (younger than six months) has been reported in the medical literature [1].

As revealed by this case, ultrasound is a valuable imaging tool for the detection of spine and spinal canal lesions in newborns and infants.
